# Clinical and radiological efficacy of spino-pelvic fixation in the treatment of neuromuscular scoliosis

**DOI:** 10.1038/s41598-023-36981-w

**Published:** 2023-06-20

**Authors:** Jae Hyuk Yang, Hong Jin Kim, Dong-Gune Chang, Yunjin Nam, Gi Seul Park, Dong Gyoon Na, Seung Woo Suh

**Affiliations:** 1 Department of Orthopedic Surgery, Korea University Anam Hospital, 73 Goryeodae-ro Seongbuk-gu, Seoul, 02841 Korea; 2 Department of Orthopedic Surgery, Spine Center, Inje University Sanggye Paik Hospital, College of Medicine, Inje University, 1342, Dongil-ro, Nowon-gu, Seoul, 01757 Korea; 3 Department of Orthopedic Surgery, Korea University Guro Hospital, 148, Gurodong-ro, Guro-gu, Seoul, 08308 Korea; 4 Geelong Clinical School, School of Medicine, Faculty of Health, Deakin University, Little Malop St, Geelong, VIC 3220 Australia

**Keywords:** Neuroscience, Diseases, Neurology, Risk factors

## Abstract

Pelvic fixation is performed to obtain proper coronal and sagittal alignment when the pelvic obliquity is more than 15º in patients with neuromuscular scoliosis (NMS). Since many NMS patients were wheelchair or bed-ridden status, there has been controversy on the effect of pelvic fixation. Therefore, the purpose of this study is to analyze the effects of pelvic fixation on correction of spinal deformity and quality of life (QoL) in NMS patients. A total of 77 NMS patients who underwent deformity correction were divided into three groups and retrospectively analyzed preoperatively, postoperatively, and at 2-year follow-up: pelvic fixation group (Group A, n = 16), fixed to S1 (Group B, n = 33), and fixed to L5 (Group C, n = 28). The correction rate of scoliosis was 60.0%, 58.0%, and 56.7% in groups A, B, and C, respectively, with no statistical difference (*P* > 0.05). The correction rate of pelvic obliquity was 61.3%, 42.8%, and 57.5% in respective groups A, B, and C, with no significance (*P* > 0.05). The correction loss of scoliosis and pelvic obliquity showed no statistical significance between three groups for 2-year follow-up (all *P*s > 0.05). There were no significant differences regarding clinical outcomes and postoperative complications among the three groups (all *P*s > 0.05). Therefore, pelvic fixation using iliac screws is not substantially influencing radiological and clinical outcomes in the patients with NMS.

## Introduction

Neuromuscular scoliosis (NMS) is a progressive spinal deformity with pre-existing neuromuscular etiology and related to a pathologic abnormality in muscle tone and imbalances, which limit mobility and remain seating status^1–5^. Pelvic obliquity (PO), defined by horizontal misalignment of the pelvis in the frontal plane, is one of the common findings as well as complex deformity of spine and hip joints in NMS^3^. The pelvic imbalance in neuromuscular conditions has effect on pelvic position, coronal, and sagittal planes, which are driven by contractures in hip flexor and adductors and weakness of the hip abductors^1^. Progressive PO with unbalanced spinal deformity has an adverse effect on sitting balance, pressure, and quality of life (QoL)^4^. Ambulation and physical function in the patients with NMS have been critically influenced on QoL by loss of sitting balances^1–3^. With the inter-relationships between spine and pelvis, the classical concepts for the treatment of NMS is to correct spino-pelvic relationship with scoliosis^1–7^. However, there has been controversy over the causes of the global spinopelvic imbalances and poor sitting postures.

The surgical goal in patients with NMS is to obtain a stable, compensated spine with the torso balanced over a leveled pelvis^2–6^. Especially, correction of PO is a major concern for non-ambulatory patients to provide proper sitting balance^3,4,7^. Pelvic fixation using iliac screws is recommended to obtain coronal and sagittal alignment of the spine in cases where PO is greater than 15º^7^. The traditional concept for pelvic fixation in NMS patients is that stable long-level fixation can be required as cantilever maneuver to NMS^7–9^. However, the introduction of thoracic pedicle screw instrumentation system provides the powerful fixation and better three-dimensional correction^1,2^. The pedicle screw instrumentation system has been developed, but several studies still supported spino-pelvic fixation with scoliosis correction when PO is greater than 15º and/or scoliosis curve is more 40º to achieve proper sitting balances^7–10^. However, extending the fusion to the pelvis in frail patients increases the technical difficulty of the procedure, pseudoarthrosis, and risk of skin ulcerations^8,9^. Therefore, pelvic fixation using iliac screws remains one of main challenging issues between clinical effectiveness in the non-ambulatory NMS patients and high complication rates. To the best of our knowledge, it is currently unknown for comparison between pelvic fixation and fixation up to lumbar region only. This study aimed to analyze the effects of pelvic fixation on radiological and clinical outcomes in the patients with NMS.

## Materials and methods

**Figure 1:**
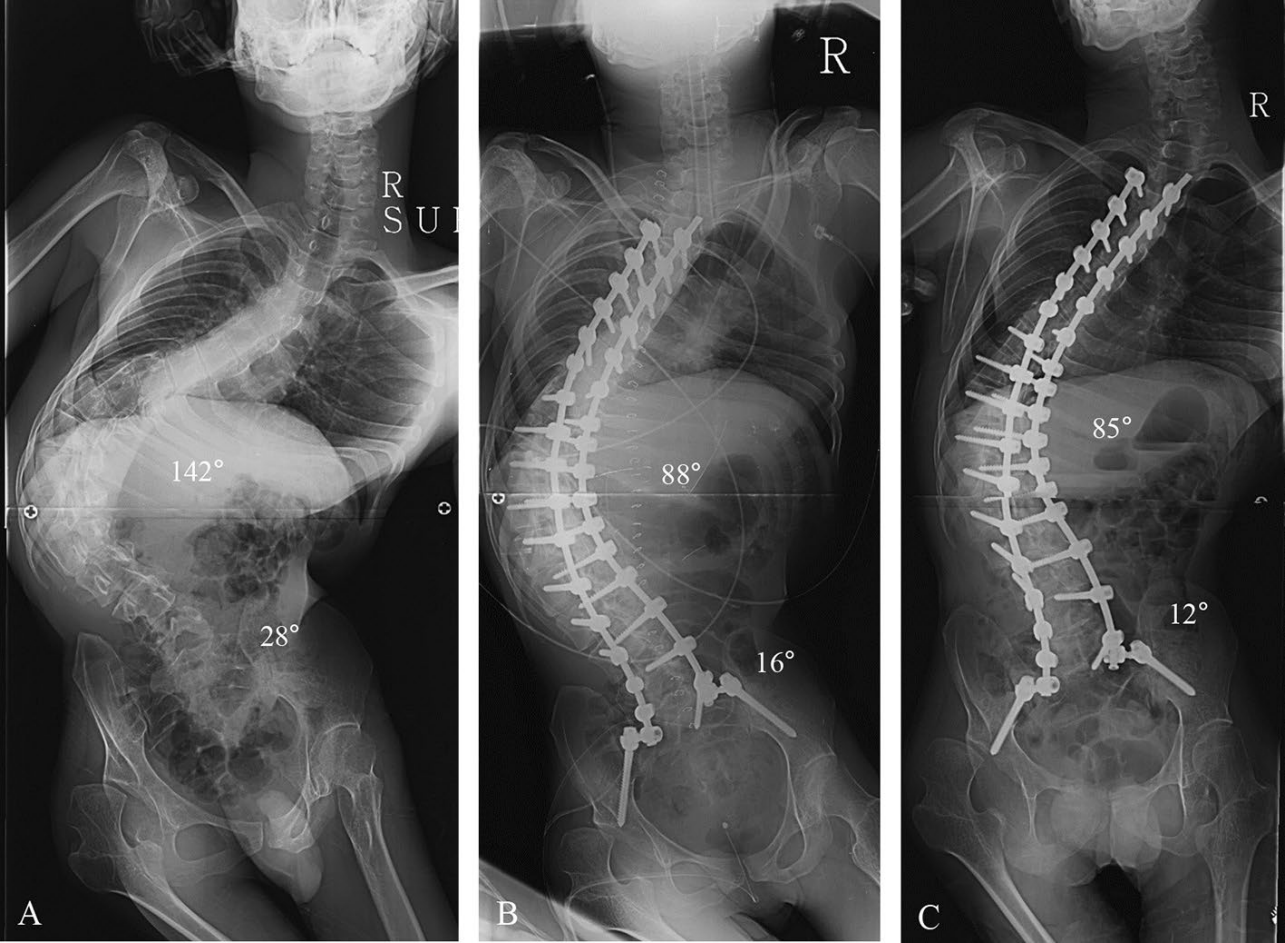
Deformity correction from T3 to spinopelvic fixation using iliac screw in patients with neuromuscular scoliosis (NMS). (**A**) A 27-year-old male patient who diagnosed with cerebral palsy (spastic quadriplegia) showed NMS. The whole-spine anterior–posterior image showed 142° of scoliosis deformity, and pelvic obliquity was 28° as measured by pelvic tilting angle. (**B**) The immediate postoperative radiograph showed 88° of the Cobb’s angle (61.4% of correction rate) and 16° of the pelvic tilting angle (42.9% of correction rate from the spinopelvic fixation). (**C**) The 2-year follow-up Cobb angle was 85° and pelvic obliquity was 12°.

**Figure 2:**
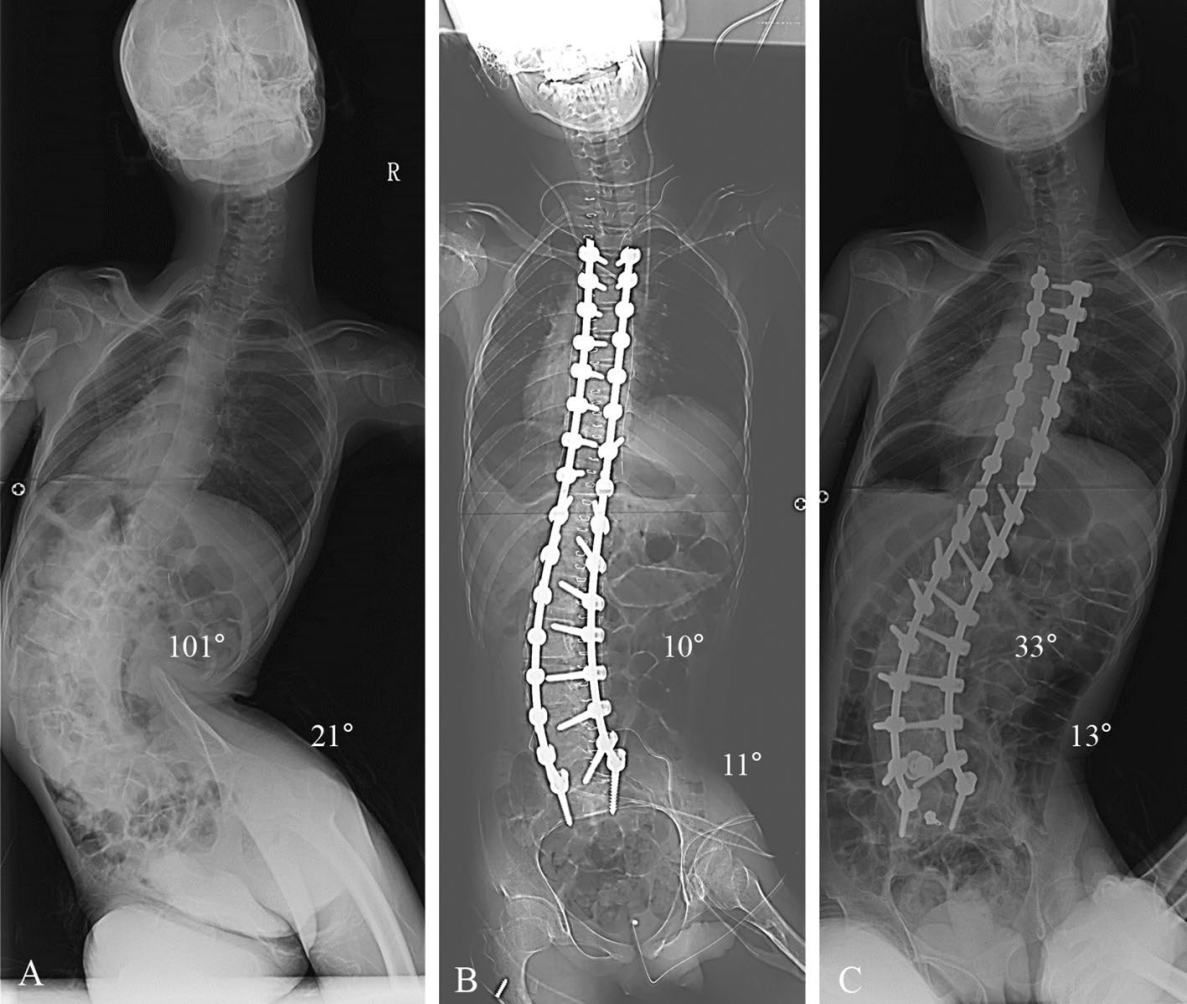
Deformity correction from T3 to S1 in patients with neuromuscular scoliosis (NMS). (**A**) A 16-year-old male patient who diagnosed with cerebral palsy (spastic quadriplegia) showed neuromuscular scoliosis. The whole-spine anterior–posterior image showed 101° of scoliosis deformity, and pelvic obliquity was 21° as measured by pelvic tilting angle. (**B**) The immediate postoperative radiograph showed 10° of the Cobb’s angle (90.1% of correction rate) and 11° of the pelvic tilting angle (47.6% of correction rate, which are not performed by spinopelvic fixation). (**C**) The 2-year follow-up Cobb angle was 33° and pelvic obliquity was 13°.

**Figure 3:**
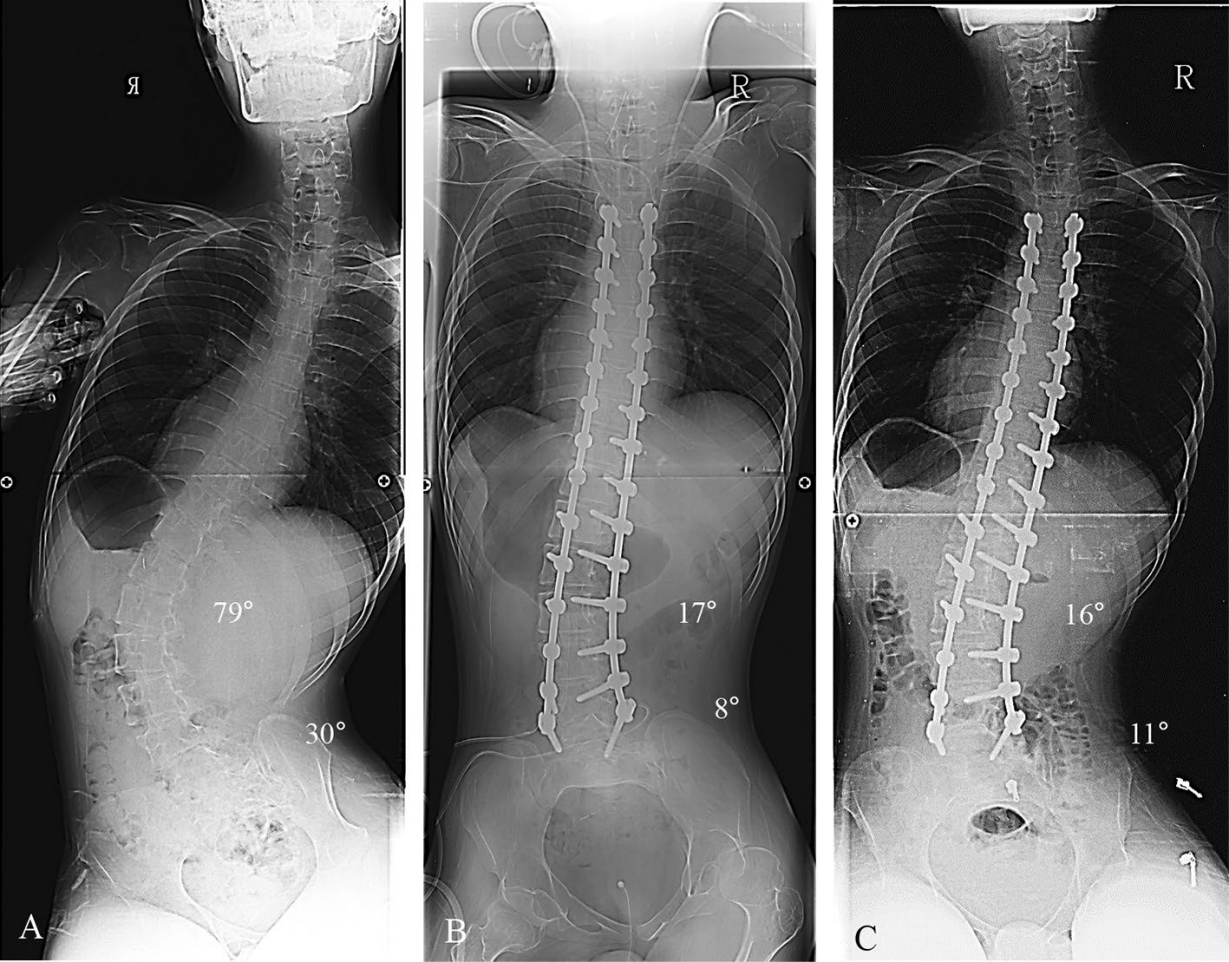
Deformity correction from T4 to L5 in patients with neuromuscular scoliosis (NMS). (**A**) A 16-year-old male patient who diagnosed with Duchenne muscular dystrophy showed neuromuscular scoliosis. The whole-spine anterior–posterior image showed 79° of scoliosis deformity, and pelvic obliquity was 30° as measured by pelvic tilting angle. (**B**) The immediate postoperative radiograph showed 17° of the Cobb’s angle (78.4% of correction rate) and 8° of the pelvic tilting angle (73.3% of correction rate, which are not performed by spinopelvic fixation). (**C**) The last follow-up Cobb angle showed 16° and pelvic obliquity was 11°.

This study was designed as retrospective, comparative analysis at a single institute where deformity correction was routinely performed. All deformity correction procedures were performed by a senior spine surgeon with vast experience in performing standard open surgeries. Considering the effect of pelvic fixation on surgical outcomes, we enrolled patients with NMS who underwent deformity correction from 2009 to 2016. Patients with Cobb’s angle less than 40º and/or PO less than 15º were excluded in our study. A total of 77 patients was included and divided into three groups as follows: the pelvic fixation group (n = 16, NMS patients who underwent pelvic fixation) (Fig. 1), fixed to S1 group (n = 33, NMS patients who underwent fixation to S1 without pelvic fixation) (Fig. 2), and fixed to L5 group (n = 28, NMS patients who underwent fixation to L5 without pelvic fixation) (Fig. 3). This study was performed after obtaining approval of the institutional review board of Korea University of Guro Hospital. The present study was performed in accordance with the contemporary amendments of the Declaration of Helsinki and within an appropriate ethical framework. Both children and parent and/or legal guardians were informed of the purpose of the study, agreed to participating, and signed informed consent for both study participation and publication of identifying information/images in an online open-access publication.

All patient data were collected from the hospital database and retrospectively analyzed. Demographic and operative variables were sex, age, body mass index (BMI), cause of NMS, hospital stay, operative time, bleeding loss, and fusion level. Radiological variables were Cobb’s angle, pelvic tilting angle, L4/pelvis angle, L5/pelvis angle, central sacral vertical line (CSVL), and sagittal vertical axis (SVA) preoperatively, postoperatively (using immediate postoperative radiographs), and at postoperative 2-year follow-up. Pelvic tilting angle was measured by methods of radiological measurements of horizontal PO as described by Osebold technique, which is easy to calculate and relatively have consistency^10^. The L4/pelvis angle was defined as the angle between the line connecting the apex of the iliac crest and the upper end of L4. The L5/pelvis angle was defined as the angle between the line connecting the apex of iliac crest and upper end of L5. All of the radiological variables were measured by two orthopedic surgeons who are very familiar with NMS surgery. The Gross Motor Function Classification System (GMFCS) was evaluated for the NMS patients with cerebral palsy etiology at preoperatively. Bridwell’s questionnaire were used for clinical evaluation preoperatively, and/or at postoperative 2-year follow-up^11^.

Statistical analysis was performed using R (version 3.3.3, The R Foundation for Statistical Computing, Vienna Austria). A normal distribution was confirmed by Kolmogorov–Smirnov test. One-way ANOVA test was used for continuous variables. For categorical variables, chi-square test and Fisher’s exact test were used for parametric data and non-parametric data, as appropriate. For variables having negative or positive values based on the measured reference point, such as CSVL and SVA, statistical comparisons of groups required converting negative numbers to positive numbers to statistically analyze differences from a reference point. Pearson’s correlation test were performed to analyze the correlations between correction amount and L4/pelvis angle, L5/pelvis angle, and pelvic tilting angle. Statistical significance was defined as *P* < 0.05.

## Results

All demographic and operative data are summarized in Table 1. The mean ages in groups A, B, and C were 19.3 years, 17.3 years and 18.0 years, with no statistical insignificance (*P* = 0.713). The etiology of NMS in our study was mostly muscular disorder (including spinal muscular dystrophy and Duchenne muscular dystrophy) and cerebral palsy. All patients with cerebral palsy in this study showed GMFCS level IV or V needing for at least wheelchair mobility. The mean hospital stays in groups A, B, and C were 27.3 days, 22.1 days, and 33.8 days, with no statistical differences (*P* = 0.304). The mean operative times in groups A, B, and C were 335.0 min, 284.1 min, and 291.1 min, with statistical difference (*P* = 0.035). The mean operative time between groups A and B showed statistical significance in post-hoc analysis by Bonferroni test. The mean fusion levels in groups A, B, and C were 15.8, 15.3, and 14.3, respectively, with statistical difference (*P* < 0.001) (Table 1).

**Table 1:** Demographic data of neuromuscular scoliosis in the three groups.

Variables	Pelvic fixation (Group A) (N = 16)	Fixed to S1 (Group B) (N = 33)	Fixed to L5 (Group C) (N = 28)	*P-*value
Sex (M:F)	13:3	20:13	16:12	0.248
Age (years)	19.3 ± 8.4*	17.3 ± 8.2*	18.0 ± 7.2*	0.713
BMI (kg/m^2^)	18.7 ± 3.4*	17.6 ± 3.2*	18.2 ± 2.9*	0.560^†^
Cause (n)				0.100^$^
SMD/DMD	11 (68.8%)	13 (39.4%)	6 (21.4%)	
Cerebral palsy	5 (31.4%)	16 (48.4%)	15 (53.7%)	
Syndromic NMDs	0	2 (6.1%)	2 (7.1%)	
Spinal cord injury	0	0	2 (7.1%)	
Others	0	2 (6.1%)	3 (10.7%)	
Hospital stay (day)	27.3 ± 24.2*	22.1 ± 12.6*	33.8 ± 43.0*	0.304
Operative time (min)	335.0 ± 65.0*	284.1 ± 66.0*	291.1 ± 63.8*	0.035
Bleeding loss (mL)	4237.5 ± 1833.7*	3275.8 ± 2552.3*	1978.6 ± 1278.1*	0.002
Fusion level	15.8 ± 0.4*	15.3 ± 1.2*	14.3 ± 1.4*	< 0.001^†^
Osteotomy	0	1	1	

*P*-values are calculated by Chi-square test.

Post-hoc analysis by Bonferroni test on 95% confidence level is performed regarding operative time.

Group A vs Group B: *P* = 0.036, Group A vs Group C: *P* = 0.103, Group B vs Group C: *P* = 1.00.

Post-hoc analysis by Bonferroni test on 95% confidence level is performed regarding bleeding loss.

Group A vs Group B: *P* = 0.369, Group A vs Group C: *P* = 0.002, Group B vs Group C: *P* = 0.044.

Post-hoc analysis by Bonferroni test on 95% confidence level is performed regarding fusion level.

Group A vs Group B: *P* = 0.516, Group A vs Group C: *P* < 0.001, Group B vs Group C: *P* = 0.005.

*N* number, *M* male, *F* female, *BMI* body mass index, *SMD* spinal muscular dystrophy, *DMD* Duchenne muscular dystrophy, *NMD* neuromuscular disorder.

*Data represent mean ± standard deviation values for each group.

^†^*P*-values are calculated by one-way ANOVA test.

^$^*P*-value is calculated by Fisher’s exact test.

Regarding radiological parameters, correction rates in groups A, B, and C were 60.0%, 58.0%, and 56.7%, respectively, with no statistical difference (*P* = 0.860). The preoperative pelvic tilt angle showed no statistical differences between group A (23.4°) and group B (24.7°) and group C (30.8°) (P = 0.094). The postoperative pelvic tilting angles in group A, B, and C were 9.8°, 12.7°, and 13.0°, respectively (*P* = 0.514). None of these differences were statistically significant in correction rate or loss of correction in pelvic tilting angle (all *P-*values > 0.05). Using the Maloney technique, the preoperative, postoperative, and 2-year follow-up pelvic tilt angle also showed no statistical difference between three groups (all *P*s > 0.05). Furthermore, there were no statistical differences in terms of L4/pelvis angle or L5/pelvis angle (all *P-*values > 0.05) (Table 2). Pearson’s correlation test was performed to evaluate the correlation between correction amount and pelvic tilting angle, L4/pelvis angle, and L5/pelvis angle. There were no statistically significant correlations between correction amount and L4/pelvis angle, L5/pelvis angle, or pelvic tilting angle in total patients, pelvic fixation patients, no pelvic fixation patients, fixed to S1 patients, and fixed to L5 patients (Table 3).

**Table 2:** Comparison of radiological parameters of the three groups.

Variables	Pelvic fixation	Fixed to S1	Fixed to L5	*P-*value
	(Group A) (N = 16)	(Group B) (N = 33)	(Group C) (N = 28)	
Cobb’s angle (°)				
Preoperative	92.5 ± 37.5	92.4 ± 23.3	95.0 ± 26.3	0.930
Postoperative	40.2 ± 29.1	40.5 ± 25.2	40.4 ± 19.1	0.999
∆ Cobb’s angle	52.4 ± 17.8	51.9 ± 19.6	54.6 ± 24.6	0.883
2-year follow-up	44.7 ± 34.3	44.1 ± 24.7	40.6 ± 23.8	0.86
Correction rate (%)	60.0 ± 18.5	58.0 ± 22.0	56.7 ± 18.3	0.871
Loss of correction	3.8 ± 12.2	5.4 ± 11.3	0.3 ± 15.9	0.413
Pelvic tilt angle (°)				
Preoperative	23.4 ± 12.1	24.7 ± 8.7	30.8 ± 16.2	0.094
Postoperative	9.8 ± 10.5	12.7 ± 8.4	13.0 ± 10.1	0.514
∆ Pelvic tilt angle	13.6 ± 9.3	12.0 ± 11.4	17.8 ± 11.9	0.137
2-year follow-up	10.9 ± 12.3	12.0 ± 8.0	15.9 ± 13.5	0.321
Correction rate (%)	61.3 ± 35.4	42.8 ± 40.2	57.5 ± 25.9	0.131
Loss of correction	0.6 ± 7.9	0.8 ± 6.2	2.3 ± 12.5	0.806
L4/pelvis angle (°)				
Preoperative	13.2 ± 10.7	12.8 ± 8.8	16.1 ± 9.4	0.358
Postoperative	9.1 ± 6.3	6.6 ± 5.3	9.2 ± 6.2	0.177
∆ L4/pelvis angle	4.1 ± 9.0	6.1 ± 7.0	7.0 ± 8.3	0.518
2-year follow-up	9.0 ± 6.7	6.4 ± 4.7	9.4 ± 6.7	0.182
L5/pelvis angle (°)				
Preoperative	15.1 ± 9.6	11.6 ± 6.6	14.7 ± 7.8	0.196
Postoperative	8.1 ± 6.2	6.9 ± 4.7	8.8 ± 5.5	0.376
∆ L5/pelvis angle	7.1 ± 8.0	4.3 ± 5.9	6.3 ± 8.5	0.382
2-year follow-up	8.1 ± 6.2	6.2 ± 4.7	8.4 ± 7.5	0.432
CSVL (mm)				
Preoperative	64.9 ± 34.7	56.9 ± 43.5	42.1 ± 29.6	0.118
Postoperative	35.3 ± 29.8	32.0 ± 24.9	38.2 ± 37.7	0.739
∆ CSVL	29.6 ± 36.8	25.0 ± 47.7	4.0 ± 36.0	0.076
SVA (mm)				
Preoperative	74.6 ± 65.4	64.9 ± 58.7	69.5 ± 54.2	0.858
Postoperative	25.5 ± 38.3	43.3 ± 36.0	41.4 ± 35.5	0.255
∆ SVA	49.1 ± 67.6	21.6 ± 69.0	28.2 ± 65.0	0.408

Data all represent mean ± standard deviation values for each group.

*N* number, *CSVL* central sacral vertical line, *SVA* sagittal vertical axis.

**Table 3:** Pearson’s correlation analysis between correction amount and L4/pelvis, L5/pelvis, and pelvic tilting angle.

	Correction amount	Preop L4/pelvis angle	∆L4/pelvis angle	Preop L5/pelvis angle	∆L5/pelvis angle	Preop pelvic tilting angle	∆Pelvic tilting angle
Total patients (N = 77)							
Correction amount	1	0.182 P = 0.112	0.131 P = 0.256	−0.096 P = 0.408	−0.028 P = 0.809	0.053 P = 0.254	0.132 P = 0.254
Pelvic fixation group (group A, N = 16)							
Correction amount	1	−0.063 P = 0.816	−0.179 P = 0.506	−0.153 P = 0.570	−0.166 P = 0.539	0.322 P = 0.223	−0.121 P = 0.655
No pelvic fixation group (group B and C, N = 61)							
Correction amount	1	0.243 P = 0.059	0.207 P = 0.109	−0.081 P = 0.534	0.004 P = 0.973	0.000 P = 0.999	0.171 P = 0.187
Fixed to S1 (group B, N = 33)							
Correction amount	1	0.104 P = 0.566	0.186 P = 0.299	0.086 P = 0.632	0.160 P = 0.375	−0.134 P = 0.456	0.157 P = 0.384
Fixed to L5 (group C, N = 28)							
Correction amount	1	0.357 P = 0.062	0.219 P = 0.262	−0.242 P = 0.215	−0.112 P = 0.571	0.045 P = 0.820	0.168 P = 0.394

No parameters were significantly correlated with correction amount.

*N* number.

Bridwell’s questionnaires were used to assess the functional outcomes among all patients. There was no significant difference in mean functional ability (*P* = 0.650), comorbidities (*P* = 0.276), or satisfaction with surgery (*P* = 0.860) among groups A, B, and C at postoperative 2-year follow-up (Table 4).

**Table 4:** Comparison of clinical outcomes in the three groups.

Variables	Pelvic fixation (Group A) (N = 16)	Fixed to S1 (Group B) (N = 33)	Fixed to L5 (Group C) (N = 28)	*P-*value
Functional ability	0.1 ± 0.6	0.1 ± 0.6	0.0 ± 0.6	0.650
Comorbidities	0.2 ± 0.5	-0.1 ± 0.5	0.1 ± 0.5	0.276
Satisfaction with surgery	0.0 ± 1.0	0.1 ± 0.7	0.1 ± 0.9	0.860

Data represent mean ± standard deviation values for each group.

*P*-values are calculated by one-way ANOVA test.

*N* number.

Regarding the complications, two patients in group A, one patient in group B, and six patients in group C were respectively experienced wound infection (*P* = 0.058). Most importantly, pedicle screw and iliac screw-related skin problem was seen in 10 (58.8%), 2 (6.1%) and 4 (14.8%) patients in groups A, B, and C (*P* < 0.001). Pulmonary complication was seen in 1, 7, and 5 patients in groups A, B, and C, respectively, with no significant difference (*P* = 0.417). Only one patient of group B experienced neurological deficits (Table 5).

**Table 5:** Comparison of postoperative complications between three groups.

Variables	Pelvic fixation (Group A) (N = 17)	Fixed to S1 (Group B) (N = 33)	Fixed to L5 (Group C) (N = 27)	*P-*value
Wound infection (n)	2 (11.8%)	1 (3.0%)	6 (22.2%)	0.058*
Skin problem by pedicle screw (n)	10 (58.8%)	2 (6.1%)	4 (14.8%)	< 0.001*
Pulmonary complication (n)	1 (5.9%)	7 (21.2%)	5 (18.5%)	0.417
Neurological complication (n)	0	1 (3.0%)	0	

Data all represent numbers for each group.

*P*-values are calculated by Chi-square test.

*N* number.

*P-values are calculated by Fisher’s exact test.

## Discussion

There have been surgical issues for non-ambulatory patients with NMS in terms of deformity correction and improvement in functional status. O’Brien et al. suggested the importance of PO corrections for non-ambulatory patients with NMS because leveling the pelvis provided basic functional abilities such as sitting and walking^12^. Correction of PO is considered standard treatment but involves the surgical risk because pelvic fixation increases surgical time, blood loss, risk of infection, and ambulatory difficulties^3,4^. Furthermore, NMS patients with PO treated conservatively required the use of a wheelchair and had only functional capacity^2^. Therefore, we analyzed the effect of pelvic fixation in NMS patients with PO in view of radiological and clinical outcomes.

In our surgery, the correction rate was comparable outcomes to those of other studies, which reported 63% correction of Cobb’s angle and 55% correction of PO^13,14^. Therefore, proper correction of spinal curve can provide sufficient correction of PO from our study. The one of the most notable point in this study was that there was no significant difference in correction rate of the main curve and PO between the three groups. Pelvic fixation was performed to achieve stability and additional force and was not associated with correction rate. That is to say, the pelvic fixation is required to the NMS patients with spinopelvic rigidity, not high degree of PO. In addition, it is necessary to reconsider the relationship between degree of PO and rigidity of spinopelvic imbalance.

Pelvic fixation in NMS is determined based on pelvic tilting angle. In the most cases, pelvic fixation is performed when the pelvic tilting angle is 15° or higher. In conventional concepts, balancing the spino-pelvic alignment facilitate postural wheelchair support and increase seating tolerances, which measured by PO. However, our study showed no difference between groups by pelvic tilting angle. In the majority of our patients, the apex of curvature was in the upper or middle lumbar vertebrae and was rarely observed from L4 to S1. From our study, L4/pelvis and L5/pelvis angles were not significant differences between three groups, which means similar level of fractional curves. Moreover, we also observed no significant correlation with pelvic tilting angle regarding L4/pelvis and L5/pelvis angle. It could be thought that the rigidity for spino-pelvic alignment in NMS may be in other factors except for radiological factors, especially skeletally immature patients. Considering our findings comprehensively, pelvic balance including pelvic angle correction is not an influential factor for main curve correction, which means that pelvic fixation using iliac screws is not an effect on radiological parameters as well as clinical outcomes. Further development that effect on pelvic rigidity may be needed in the case of skeletal immature NMS patients.

A one prospective cohort study by Mazda et al. provide the similar results with our data. They also suggest the pelvic fixation was not the mandatory and did not affect revision rate or clinical outcomes^15^. Compared to the study from Mazda et al., the age in the study group is relatively higher in our study. The flexibility of the spine and pelvis decrease along with increased age. Even in this relative old age group, our important findings suggest that the effectiveness of the pelvic fixation is insufficient in the patients with NMS. Therefore, the surgical indication of pelvic fixation needs to be reconsidered for the NMS patients.

In surgical cases of preserved mobile segments, the patients maintain relative flexibility and able to hold a sitting position^4,16^. However, Akesen et al. reported that spinopelvic fusion with iliac screws in neuromuscular scoliosis did not increase implant failure and helped achieve better functional results^17^. Tsirikos et al. also studied that the effect of pelvic fixation in NMS patients caused by spastic cerebral palsy. For 24 ambulatory patients, spinopelvic fixation preserved their ambulatory functions for 2.86 years for follow-up. Our findings are contrast to their results^18^. We found no significance differences between the three groups, indicating that spinopelvic fusion with iliac screws did not improve spinal correction rate or patients or patient care takers-relatives’ QoL. Therefore, fusion without pelvic fixation could be beneficial to preserve the mobility of segments, providing flexibility needed for sitting. Judging from these results, it is necessary to reconsider the indication of spinopelvic fixation in the patients with NMS.

The surgical complication rate in NMS is high due to relatively poor nutrition and associated cardiopulmonary complications^8^. Especially, pelvic fixation results in greater blood loss, longer operative time, and need for more extensive soft tissue dissection^19^. Pelvic fixation also increases vulnerability to infection and screw-related skin problems because the fusion for NMS was required to long-level fixation^14–16^. Our data showed group A detected highest complication rate of screw-related skin problem, which are one of the reasons to decision-making of no spinopelvic fixation in this study. Although the decision-making of fixation level is determined by the surgeon’s preference from time sequence based on surgical experience, out data showed spinopelvic fixation in NMS patients need re-evaluated from considering the risk and benefits.

Infection rates in NMS range from 6 to 19% in the literatures, which is comparable to that in our study^20^. Patients with cerebral palsy were associated with postoperative infection because of poor wound healing, incontinence, and impaired communication^14,20,21^. Cerebral palsy in NMS showed a 9.0% infection rate in our study, comparable to the 6.1 to 8.7% in the literature^22^. The largest proportion of wound infection in our study was seen in group C (fixation to L5) with cerebral palsy, allowing greater mobility. Therefore, fixation to L5 in NMS patients with cerebral palsy requires additional caution to avoid deep wound infection.

Large curvature greater than 65° was associated with reduced lung volume, which can result in ventilation/perfusion mismatch^8^. Kang et al. reported preoperative Cobb’s angle greater than 69° and age older than 16.5 years as predictors for postoperative pulmonary complications in NMS^22^. However, they included little information of pulmonary complication rate according to fusion level. In our study, the fusion level was not associated with pulmonary complications.

This study had some limitations. The sample size was relatively small, and the study design was retrospective. However, our included group which the NMS patients who underwent deformity correction is a relatively rare case. Furthermore, the collected data is not collected by proper indication of ‘not pelvic fixation’. Future trials may be needed to re-evaluate the indication of pelvic fixation in the patients with NMS. The age group was relatively old compared to existing study so it is not enough to show the representation of all NMS patients’ group^15^. Our study focused on the effectiveness of spinopelvic fixation compared to lumbar or lumbosacral fixation. The relatively short follow-up period is also considered a limitation of our study. However, this study was not suggested the determination of distal fusion level in NMS patients as one of limitation. In addition, mechanical complications of screw and/or rod breakage were not considered in our study. Large multicenter studies with long-term follow-up are needed to support our results.

## Conclusions

Pelvic fixation does not affect coronal or sagittal correction as well as QOL in neuromuscular scoliosis. Pelvic fixation using iliac screws is not substantially influenced on radiological and clinical outcomes in the patients with NMS. Therefore, the surgical decision of pelvic fixation should be carefully reconsidered in NMS patients.

## Data Availability

The datasets generated and/or analysed during the current study are not publicly available since the data is available only to institutions authorized by Institutional Review Board, but are available from the corresponding author on reasonable request.
